# Supplementary material for: Chemical Composition and Biological Activity of Argentinian Propolis of Four Species of Stingless Bees
Source: Molecules. 2022 Nov 9;27(22):7686. doi: 10.3390/molecules27227686 (PMC9697202; doi:10.3390/molecules27227686)
Supplement: Supplementary file 1 [file molecules-27-07686-s001.zip › molecules-2008342-supplementary.pdf]

Supplemeent of

Chemical composition and biological activity of Argentinian propolis of four species of stingless bees

Valery A. Isidorov, Jolanta Maslowiecka, Lukasz Szoka, Naldo Pellizzer, Dora Miranda, Ewa Olchowik-Grabarek, Monika Zambrzycka, Izabela Swiecicka

Correspondence to: Valery A. Isidorov (Isidorov@uwb.edu.pl)

Table S1. Chemical composition of the volatile components of propolis Argentine stingless bees. 1 - *Scaptotrigona* aff. *postica;*2 - *Tetragona clavipes*; 3 - *Melipona quadrifasciata quadrifasciata*; 4 - *Tetragonisca fiebrigi*

Designations: *m/z* is the mass number of the most intense peaks in the mass spectrum of the compound (in descending order of intensity); M^+ *is the mass number of the molecular peak of the compound;* RI^{Calc} is the calculated value of the retention index*;* RI^{Lit} is the literary value of the retention index.

Compound	CAS	*m/z*	M^+	RI^{Calc}	RI^{Lit}	1	2	3	4
Acetaldehyde	75-07-0	29,44,43,42	44	-	432	-	-	0.06	-
Ethanol	64-17-5	31,45,29,46	46	-	484	3.46	1.70	1.06	8.35
Acetone	67-64-1	43,58,42,39	58	-	501	-	-	1.47	-
Propanol	73-21-8	31,29,42,69	60	-	546	1.95	0.97	-	1.22
n-Hexane	110-54-3	57,43,41,29	86	600	600	-	-	0.25	1.40
Ethyl acetate	141-78-6	43,61,45,70	88	611	606	4.30	5.31	0.41	6.83
Methyl propionate	554-12-1	29,57,88,59	88	625	625	1.86	trace	-	-
1-Butanol	71-36-3	56,31,41,43	-	652	652	-	-	0.08	-
Acetic acid	64-19-7	43,45,60,42	60	659	645	7.60	2.68	0.25	12.24
n-Heptane	142-82-5	57,43,41,71	100	700	700	-	-	0.09	-
Ethyl propionate	105-37-3	29,57,102,75	102	710	714	6.60	2.87	-	2.67
Methyl cyclohexane	108-87-2	83,55,98,41	98	719	722	-	-	0.02	-
Isopentanol	123-51-3	55,42,43,41	-	731	734	1.98	1.10	0.03	1.05

Propanoic acid	79-09-4	74,45,73,29	74	740	738	1.10	1.33	-	0.24
Isopropyl propionate	637-78-5	57,43,75,101	-	750	750	-	trace	-	-
Pyridine	110-86-1	79,52,78,51	79	755	754	-	2.70	0.73	-
Toluene	108-88-3	91,92,65,39	92	762		-	-	1.07	0.80
n-Octane	111-65-9	57,71,43,85	114	800	800	-	-	-	0.41
Ethyl butanoate	105-54-4	71,43,88,29	116	803	809	-	0.83	-	2.63
Propyl propionate	106-36-5	57,75,29,43	-	811	811	5.14	1.90	-	
Ethyl lactate-1	97-64-3	45,29,75,43	-	814	815	-	-	-	0.33
Ethyl lactate-2	N/A	45,29,75,43	-	816	-	-	-	-	0.34
Butyric acid	107-92-6	60,73,43,41	88	822	800	-	0.43	-	-
Ethyl 2-methylbutanoate	7452-79-1	57,102,85,41	130	848	847	0.11	trace	-	0.13
Ethyl 3-methylbutanoate	108-64-5	88,57,85,29	130	851	850	0.07	0.19	-	0.19
Isobutyl propionate	540-42-1	57,29,87,56	-	864	866	0.19	0.07	-	-
p-Xylene	106-42-3	91,106,105,77	106	867	866	-	-	0.06	-
1-Hexanol	11-27-3	56,43,55,41	-	869	870	0.43	-	-	0.63
Isopentyl acetate	123-92-2	43,55,70,61	-	874	874	0.09	0.07	-	0.85
Cyclofenchene	488-97-1	93,92,121,136	136	878	884	-	0.07	0.04	-
2-Methybutan-1-ol acetate	624-41-9	43,70,55,73	-	878	877	-	-	-	0.27
Styrene	100-42-5	104,103,78,51	104	890	893	1.65	1.39	6.46	0.68
2-Heptanol	543-49-7	45,55,43,41	-	902	904	0.47		-	0.18
Bornylene	464-17-5	93,121,136,108	136	903	900	-	trace	1.24	-
n-Propyl butanoate	105-66-8	43,71,89,41	-	901	896	-	0.89	-	-
Ethyl pentanoate	539-82-2	85,88,57,60	130	903	902	-	-	-	0.33
n-Butyl propionate	590-01-2	57,56,75,41	-	908	908	0.24	0.49	-	-
Butyrolactone	96-48-0	42,41,86,28	86	916	916	-	-	-	1.99
Tricyclene	508-32-7	93,91,79,121	136	922	920	-	0.81	1.29	-
α-Thujene	2867-05-2	93,92,91,77	136	925	926	-	-	-	0.20

α-Pinene	80-56-8	93,91,77	136	930	932	1.98	12.16	20.97	5.34
Ethyl 3-hydroxybutyrate	54074-85-0	71,117,43,89	-	934	937	-	-	-	0.32
Camphene	79-92-5	93,121,91,136	136	944	946	0.08	2.63	3.29	-
Verbenene	4080-46-0	119,134,41,43	134	952	955	-	0.66	0.49	-
γ-Valerolactone	108-29-2	56,41,85,43	100	953	953	-	-	-	0.09
Benzaldehyde	100-52-7	106,105,77,51	106	960	962	-	-	0.16	-
Isopentyl propionate	105-68-0	57,70,55,43	-	969	969	0.48	-	-	-
3,7,7-Trimethyl-1,3,5-cycloheptatriene	3479-89-8	119,91,77,41	134	970	970	-	-	0.40	-
β-Pinene	127-91-3	93,41,69,39	136	979	982	0.52	8.57	10.10	1.96
Myrcene	123-35-3	41,93,69,39	136	993	991	0.12	2.19	4.29	0.40
Phenol	108-95-2	94,66,65,39	94	996	991	0.17	-	-	-
Menthene 3	619-52-3	95,81,123,138	138	998	990	-	0.57	-	-
Ethyl hexanoate	123-66-0	88,43,99,29	144	1002	1003	0.32	-	-	1.26
α-Phellandrene	99-83-2	93,77,92,91	136	1004	1004	-	0.79	0.50	-
3-Carene	13466-78-9	93,91,121,92	136	1010	1011	3.17	2.35	3.93	4.57
Hexyl acetate	142-92-7	43,56,55,84	-	1014	1014	0.33	-	-	0.24
α-Terpinene	99-86-5	121,93,136,91	136	1017	1015	-	1.23	1.42	0.12
p-Cymene	99-87-6	119,134,91,118	134	1023	1024	0.09	6.85	5.70	028
β-Phellandrene	555-10-2	93,77,136,91	136			-	1.77	-	-
Limonene	138-86-3	68,93,67,136	136	1028	1028	0.25	1.77	6.74	0.81
2-Ethylhexan-1-ol	104-76-7	57,43,41,55	-	1033	1032	27.4	trace	3.58	17.14
Benzyl alcohol	100-51-3	79,77,108,107	108	1037		-	-	0.24	0.32
o-Cymene	527-84-4	119,91,117,134	134	1039	1039	-	-	0.21	-
cis-β-Ocimene	3338-55-4	93,79,41,91	136	1039	1045	-	0.32	0.10	-
2-Heptanol acetate	5921-82-4	43,41,56,87	-	1044	-	-	0.04	-	-
trans-β-Ocimene	3779-61-1	93,79,91,41	136	1049	1048	-	0.24	0.19	-
γ-Terpinene	99-85-4	93,136,91,121	136	1058	-	-	1.37	1.37	0.25

trans-Sabinene hydrate	17699-16-0	93,121,91,77	-	1066	1066	-	0.18	0.10	-
Monoterpenoid C10H16O	-	93,91,77,41	152	1071	-	-	-	0.02	-
1-Octanol	111-87-5	56,55,41,43	-	1073	1072	-	0.06	-	-
trans-Linalool oxide	34995-77-2	59,94,43,111	-	1073	1073	0.16	-	-	0.19
p-Cymenene	1195-32-0	117,132,91,115	132	1082	1089	-	-	0.04	-
Terpinolene	586-62-9	93,121,136,91	136	1089	1088	-	3.18	3.55	0.32
Guaiacol	90-05-1	109,124,81,53	124	1090	1089	0.19	-	-	-
cis-Sabinene hydrate	N/A	93,121,92,91	-	1097	1098	-	0.18	0.17	-
Ethyl heptanoate	106-30-9	88,113,101,60	-	1100	1100	0.13	-	-	0.66
Linalool	78-70-6	71,93,55,43	154	1101	1100	-	0.26	0.11	-
2-Nonanol	628-99-9	45,69,41,55	-	1102	1103	-	trace	-	0.12
endo-Fenchol	14575-74-7	81,80,43,69	154	1112	1113	-	0.45	0.36	-
2-Phenyl ethanol	60-12-8	91,92,122,65	122	1114	1112	0.08	-	-	0.32
cis-*p*-Menth-2-en-1-ol	29803-82-5	43,93,139,121	154	1120	1121	-	0.18	0.16	-
α-Campholenal	4501-58-0	108,93,95,152	152	1120	1121	-	-	0.16	-
Methyl octanoate	111-11-5	74,87,127,43	158	1127	1127	-	0.08	-	-
Nopinone	24903-95-5	83,55,95,41	138	1134	1136	-	-	0.24	-
trans-Pinocarveol	547-61-5	92,70,55,83	152	1136	1140	-	0.82	0.61	-
Camphor	464-48-2	95,81,41,108	152	1141	1144	-	0.35	0.47	-
trans-Verbenol	1820-09-3	109,41,94,81	152	1144	1145	-	0.36	0.55	-
2-Ethylhexyl acetate	103-09-3	43,70,57,55	-	1152	1152	7.67	0.22	0.10	4.62
Pinocamphone	547-60-4	55,69,83,41	152	1158	1161	-	0.34	0.43	-
Pinocarvone	30460-92-5	108,81,53,150	150	1160	1160	-	-	0.16	-
Borneol	507-70-0	95,110,139,41		1164	1166	-	0.72	0.45	-
Isopinocamphone	15358-88-0	55,69,83,41	152	1172	1175	-	0.92	0.78	-
1-Nonanol	143-08-8	56,55,70,43	-	1174	1174	0.02	-	-	-
4-Terpineol	562-74-3	71,93,111,154	154	1177	1177	-	2.10	1.83	-

Benzoic acid	65-85-0	105,122,77,51	122	1179	1183	-	???	-	-
Diethyl succinate	123-25-1	101,129,29,55	174	1185	1188	-	-	-	0.49
p-Cymen-8-ol	1197-01-9	43,135,132,91	150	1185	1186	-	0.28	0.26	-
α-Terpineol	98-55-5	93,59,121,136	-	1190			0.66	2.19	-
Myrtenal	564-94-3	79,107,108,91	150	1194	1195	-	-	0.24	-
NN	-		-	1195	-	0.07	-	-	-
Myrtenol	515-00-4	79,91,108,41	152	1195	1198	-	0.32	0.40	-
Methyl chavicol (estragol)	140-67-0	148,147,121,117	148	1198	1198	-	-	1.14	-
Ethyl octanoate	106-32-1	88,101,127,60	172	1199	1198	0.07	0.93	-	0.94
Monoterpenoid C10H16O			152	1200	-	-	-	0.20	-
Verbenone	1196-01-6	107,135,91,80	150	1207	1210	-	0.27	0.59	-
Monoterpenoid C10H18O	-		154	1216	-	-	-	0.05	-
trans-Carveol	1197-07-5	109,84,119,41	152	1219	1219	-	-	0.07	-
α-Fenchyl acetate (endo-)	1351-11-1	43,81,80,136	-	1219	1221	-	0.05	-	-
Thymol, methyl ether	1076-56-8	149,164,91,119	164	1236	1241	-	0.25	0.18	-
Carvacrol methyl ether	6379-73-3	149,164,119,91	164	1245	1244	-	-	1.98	-
Piperitone	89-81-6	82,110,95,137	152	1253	1255	-	0.09	-	-
Linalyl acetate	115-95-7	93,43,41,69	-	1258	1257	-	0.30	-	-
Bornyl acetate	5655-61-8	95,93,121,136	196	1285	1287	-	0.67	0.08	-
Propyl octanoate	624-13-5	43,145,127,61	186	1294	1292	-	0.17	-	-
4-Terpineol acetate	4821-04-9	93,121,136,91	-	1300	1300	-	0.09	0.09	-
Myrtenyl acetate	1079-01-2	93,119,43,92	-	1326	1327	-	0.05	-	-
α-Terpenyl acetate	80-26-2	121,43,93,136	-	1341	1349	-	0.07	0.05	-
α-Cubebene	17694-14-8	161,119,105,81	204	1350	1350	0.10	1.30	0.63	-
Ylangene	14912-44-8	105,93,119,161	204	1371	1372	-	0.16	0.05	-
α-Copaene	3856-25-5	161,119,105,93	204	1376	1377	0.31	0.73	0.24	trace
β-Bourbonene	5208-59-3	81,80,123,161	204	1384	1387	-	0.42	0.12	-

Diethyl adipate	141-28-6	157,111,115,128	202	1392	1390	-	-	-	0.29
β-Elemene	515-13-9	81,93,68,107	204	1393	1392	0.06	0.41	-	
Ethyl decanoate	110-38-3	88,101,73,43	200	1401	1398		trace	-	trace
Longofolene	475-20-7	161,94,93,91	204	1403	1407		0.37	0.54	-
α-Gurjunene	489-40-7	204,189,161,105	204	1410	1409	2.55	-	-	5.14
β-Funebrene	N/A	119,41,161,91	204	1411	1412	-	0.34	0.34	0.14
β-Caryophyllene	87-44-5	93,133,91,79	204	1420	1418	1.41	3.02	0.45	2.83
β-Copaene	N/A	161,105,91,41	204	1429		-	0.30	0.04	-
NN	-		-	1433	-	-	0.08	-	-
Aromadendrene	489-39-4	41,161,91,93	204	1438	1440	-	0.37	-	-
$C_{15}H_{24}$	-	93,161,91,41	204	1447	-	-	0.23	-	-
α-Humulene	6753-98-6	93,121,80,41	204	1452	1454	0.40	0.83	0.08	0.98
Alloaromadendrene	25246-27-6	161,91,41,93	204	1459	1464	0.16	-	-	-
cis-Muurola-4(14),5-diene	N/A	161,105,119,81	204	1462	1465		0.32	-	0.26
γ-Gurjunene	22567-17-5	161,81,105,107	204	1471	1473	0.06	-	-	trace
trans-Cadina-1(6),4-diene	N/A	189,161,204,105	204	1473	1476		trace	-	-
Selina-4,11-diene	17066-67-0	41,204,105,107	204	1474	1477	-		-	0.21
γ-Muurolene	30021-74-0	161,204,105,119	204	1477	1480	-	0.91	0.13	-
Germacrene D	23986-74-5	161,105,91,119	204	1479	1480	-	1.49	-	-
β-Selinene	17066-67-0	41,204,105,93	204	1484	1484	0.47	-	0.06	1.28
α-Selinene	473-13-2	189,204,93,81	204	1493	1496	0.43		0.06	1.41
Bicyclogermacrene	67650-90-2	121,93,41,107	204	1494	1499	-	1.03	-	-
α-Muurolene	10208-80-7	105,161,204,94	204	1499	1500	-	0.41	0.09	-
γ-Cadinene	39029-41-9	161,204,119,105	204	1516	1517	-	0.72	0.08	-
δ-Cadinene	483-76-1	161,134,105,119	204	1525	1527	0.09	1.19	0.23	trace
trans-Cadina-1,4-diene	N/A	161,119,105,93	204	1531	1536	-	0.05	-	
α-Cadinene	24406-05-1	105,161,204,93	204	1536	1540	-	0.08	-	

α-Calocorene	21391-99-1	157,142,200,115	200	1541	1546	-	0.04	-	
Sesquiterpenoid C15H26O	-	161,41,93,119	222	1564	-	-	0.05	-	
Spathulenol	6750-60-3	43,119,205,41	220	1575	1580	-	0.13	-	
Caryophyllene oxide	1139-30-6	43,41,79,93	220	1580	1586	-	0.10	-	
Cedrol	77-53-2	95,150,151,43	222	1597	1600	-	0.08	0.08	
Humulene II epoxide	19888-34-7	43,109,138,41	-		1606	-	0.02	-	
Junenol	472-07-1	109	222		1620	-	0.24	-	
Eremoligenol	10219-71-3		222		1630	-	0.05	-	
Sesquiterpenol C15H24O acetate	-	43,119,189,105	262			-	0.06	-	
α-Cadinol	481-34-5	43,95,121,204	222		1658	-	0.07	-	

Table S2. Chemical composition (% TIC) of ether extracts of Argentine propolis from stingless bees. 1 - *Scaptotrigona* aff. *postica;* 2 - *Tetragona clavipes*; 3 - *Melipona quadrifasciata quadrifasciata*; 4 - *Tetragonisca fiebrigi*

						The stingless bees			
Compound	CAS	m/z	M^+			1	2	3	4
β-Thujene	2867-05-2	93,77,91	136	927	926	-	trace	0.11	-
α-Pinene	80-56-8	93,91,77	136	932	932	-	0.54	1.89	-
Ethylamine, di-TMS	2477-39-6	174,100,73	189	965	966	-	0.86	1.05	-
Sabinene	3387-41-5	93,41,91	136	974	973	-	0.03	0.16	-
β-Pinene	127-91-3	93,41,69	136	976	975	-	0.29	0.73	-
Myrcene	123-35-3	41,93,69	136	991	991	-	-	trace	-
α-Terpinene	586-62-9	93,121,136	136	1113	1115	-	-	trace	-
p-Cymene	99-87-6	119,134,91	134	1024	1022	-	0.06	-	-
Limonene	138-86-3	93,68,91	136	1028	1028	-	-	0.09	-
β-Phellandrene	555-10-2	93,77,91	136	1030	1029	-	0.09	-	-
Lactic acid, di-TMS	17596-96-2	73,117,147	-	1076	1074	-	0.03	-	0.12
Myrtenal	564-94-3	79,107,77	150	-	1095	-	-	trace	-

Sabinene hydrate, TMS	N/A	211,73,183	226	1205	1206	-	-	0.05	-
Borneol, TMS	37555-29-6	95,73,108	226	1227	1226	-	0.03	0.07	-
(*E*)-Verbenol, TMS	N/A	73,144,119	224	1236	1235	-	trace	0.22	-
Carvacrol, methyl ether	6379-73-7	149,164,91	164	1244	1244	-	-	trace	-
Benzoic acid,TMS	2078-12-8	105,179,77	194	1247	1246	trace	trace	-	-
Octanoic acid, TMS	55494-06-9	201,75,73	216	1268	1266	-	0.07	-	-
Pinocarveol, TMS	N/A	155,73,91	224	1269	1272	-	-	0.18	-
Camphene hydrate, TMS	N/A	73,93,158	226	1282	1287	-	0.06	0.16	-
Bornyl acetate	76-49-3	95,43,121	196	1287	1287	-	trace	-	-
H3PO4, tri-TMS	10497-05-9	299,73,300	314	-	-	trace	-	-	-
Glycerol, tri-TMS	6787-10-6	73,205,147	-	1289	-	trace	-	-	0.13
Myrtenol, TMS	N/A	73,91,93	224	1298	1293	-	trace	0.15	-
Succinic acid, di-TMS	40309-57-7	147,73,75	262	1322	1324	-	-	-	trace
α-Terpineol, TMS	57304-99-1	131,73,136	226	1323	1323	-	0.03	0.21	-
Bicycloelemene	32531-56-9	121,93,79	204	1335	1339	-	trace	0.07	-
α-Copaene	3856-25-5	161,119,105	204	1375	1376	-	0.04	-	-
β-Bourbonene	5208-59-3	81,80,123	204	1385	1388	-	trace	-	-
β-Cubebene	17699-14-8	161,195,91	204	1390	1392	-	trace	-	-
Longifolene	475-20-7	161,105,91	204	1400	1404	-	-	trace	-
α-Gurjunene	489-40-7	161,105,204	204	1404	1409	trace	-	-	-
Myrtenoic acid, TMS?	N/A	73,179,105	238	1409	-	-	-	trace	-
β-Caryophyllene	87-44-5	93,91,133	204	1414	1417	trace	0.32	-	-
α-Humulene	6753-98-6	93,41,80	204	1450	1454	-	0.08	-	-
Decanoic acid, TMS	55494-15-0	229,117,73	-	1459	1461	-	0.06	-	-
γ-Muurolene	30021-74-0	161,105,204	204	1472	1480	-	0,06	-	-
D-Germacrene	23986-74-5	161,105,41	204	1482	1480	-	0.36	-	-
Bicyclogermacrene	67650-90-2	93,41,121	204	1490	1499	-	0.11	-	-
α-Muurolene	31983-22-9	105,161,94	204	1495	1502	-	trace	-	-
γ-Cadinene	39029-41-9	161,204,105	204	1512	1516	-	0.09	-	-
δ-Cadinene	483-76-1	161,134,204	204	1528	1527	-	0.17	-	-
NN (terpenoid TMS?)	-	131,73,181	224	1578	-	-	-	0.08	-

Caryophyllene oxide	1139-30-6	43,41,79	220	1578	1582	-	0.04	-	-
1-*epi*-Cubebol, TMS?	N/A	279,73,161	294	1625	1617	-	0.05	-	-
Spathulenol, TMS	N/A	143,73,202	292	1670	1674	trace	0.23	-	-
Sesquiterpenol C15H26-OTMS	-	73,143,189	294	1684	-	trace	0.11	-	-
Sesquiterpenol C15H26-OTMS	-	143,73,161	294	1695	-	-	0.04	-	-
Acorenol, TMS	N/A	131,73,75	294	1717	1720	-	0.05	-	-
Sesquiterpenol C15H26-OTMS	-	143,144,73	294	1738	-	-	-	0.16	-
α-Cadinol, TMS	N/A	143,73,161	294	1745	1747	-	0.25	-	-
β-Eudesmol, TMS	N/A	131,73,75	294	1753	1753	-	0.05	-	-
Oplopanone, TMS?	N/A	225,73,143	310	1831	-	-	0.03	-	-
Tetradecanoic acid, TMS	18603-17-3	73,117,75	300	1849	1855	trace	0.04	-	-
NN	-	131,73,75	-	1944	-	-	0.08	-	-
Manoyl oxide	596-84-9	275,257,81	290	1991	1992	-	0.06	0.06	-
Ethyl hexadecanoate	628-97-7	88,101,42	284	1992	1992	-	-	-	trace
3-Hydroxymyristic acid, di-TMS	136788-85-7	73,233,147	-	2040	2045	trace	-	-	-
Hexadecanoic (palmitic) acid, TMS	55520-89-3	117,132,73	328	2052	2052	0.12	0.15	-	0.21
Dehydroabietane	19407-28-4	255,173,159	270	2052	2057	-	-	0.15	-
NN	-	143,73,81	-	2053	-	-	-	0.13	-
Abietadiene	35241-40-8	272,257,106	272	2081	2083	-	trace	-	-
Diterpenoid, TMS	-	143,73,81	-	2089	-	-	-	0.09	-
γ-Palmitolactone	730-46-1	85,43,55	254	2100	2105	trace	-	-	-
Diterpenoid, TMS	-	73,121,120	364	2112	-	-	0.05	-	-
Diterpenoid (13-epimanool?), TMS	N/A	143,73,75	362	2126	-	-	0.22	0.94	-
Diterpenoid C20H30O	-	123,91,107	286	2154	-	-	0.04	0.10	-
NN	-	156,73,131	-	2174	-	-	-	0.09	-
Sandaracopimarinal	3855-14-9	91,123,81	286	2174	2184	-	0.06	-	-
NN	-	134,73,119	-	2187	-	-	0.08	-	-
NN	-	73,121,75	-	2194	-	-	0.03	-	-
Diterpenoid C20H30O	-	81,79,91	286	2208	-	-	0.34	0.45	-
Diterpenoid, TMS	-	73,257,81	360	2215	-	-	-	0.24	-
Linoleic acid, TMS	56259-07-5	73,75,67	352	2215	2215	0.04	-	-	0.06

Oleic acid, TMS	21556-26-3	73,75,117	354	2216	2221	0.12	0.21	-	0.22
NN	-	73,143,43	-	2222	-`	-	-	0.17	-
Diterpene acid, TMS	-	73,146,159	374	2230	-	-	-	0.40	-
Diterpenoid C20H30O	-	91,92,133	286	2226	-	-	0.12	-	-
Diterpenoid, TMS	-	73,159,257	374	2231	-	-	0.20	-	-
3-Hydroxypalmitic acid, di-TMS	136788-86-8	73,233,147	-	2233	2237	0.07	-	-	-
Octadecanoic (stearic) acid, TMS	18748-91-9	117,73,132	356	2246	2248	0.03	-	-	trace
Sempervirol, TMS	?	73,343,344	358	2237	2238	-	-	0.21	-
2,3-Dehydroferrugenol, TMS?	N/A	73,274,356	356	2238	-	-	1.20	-	-
Diterpenoid, TMS	-	73,255,81	360?	2239	-	-	-	0.18	-
Diterpene acid, TMS	-	73,121,135	374	2244	-	-	0.26	0.27	-
Ferruginol, TMS	N/A	73,358,343	358	2251	2250	-	0.90	0.45	-
Diterpenoid	-	159,173,269	284	2258	-	-	-	0.07	-
Dehydroabietal-like diterpenoid C20H28O	-	284,269,173	284	2259	-	-	0.11	-	-
Diterpenoid?	-	73,121,43	364?	2265	-	-	0.14	0.35	-
(*Z*)-9-Tricosene	27519-02-4	97,55,83	322	2271	2275	0.06	-	-	--
Diterpenoid, TMS	-	73,81,103	372	2277	-	-	0.53	0.35	-
(*Z*)-7-Tricosene	N/A	97,83,55	322	2278	-	trace	-	-	-
Copalool, TMS	N/A	73,95,257	-	2282	2286	-	0.15	0.77	-
Pimaric acid, TMS	21414-46-0	73,121,257	374	2295	2301	-	1.34	2.20	trace
n-Tricosane	638-67-5	57,71,43	324	2300	2300	0.06	-	-	-
Diterpenoid, TMS	-	143,10973	-	2303	-	-	-	0.31	-
Sandaracopimaric acid, TMS	N/A	73,121,359	374	2313	2318	-	1.17	1.10	trace
(*E*)-Communic acid, TMS	N/A	73,81,119	374	2326	2324	-	5.32	5.90	-
Isopimaric acid, TMS	21414-47-1	73,256,241	374	2331	2334	trace	4.11	5.79	0.11
Totarol, TMS	N/A	343,247,73	358	2334	2332	-	2.83	5.99	trace
Diterpene acid, TMS	-	73,257,359	374	2341	-	-	0.09	-	-
Palustric acid, TMS	N/A	241,73,240	374	2352	2354	-	4.65	1.77	trace
Diterpene acid, TMS	-	73,91,257	374	2359	2369	0.02	-	-	-
Neoabietal	19898-57-8	135,286,148	286	2363	2372	-	0.29	-	-
Isopimara-8,15-dienoic acid, TMS	N/A	73,239,256	374	2373	2376	-	2.67	1.35	trace

Methylabietate	127-25-3	43,121,316	316	2377	2385	-	0.05	-	-
Dehydroabietic acid, TMS	21414-49-3	239,73,357	372	2386	2389	0.01	3.10	8.10	0.08
Diterpenoid, TMS?	-	143,73,81	-	2395	-	-	0.45	2.13	0.62
Abietic acid, TMS	21414-50-6	256,241,73	374	2417	2416	trace	6.43	2.23	0.13
(11*E*)-Eicosenoic acid, TMS	N/A	73,75,129	382	2419	2418	-	-	-	0.23
Diterpenoid, TMS	-	135,73,261	-	2426	-	-	0.27	-	-
3-Hydroxystearic acid, di-TMS	N/A	73,147,233	-	2430	2428	0.02	-	-	-
13-*epi*-Cupressic acid, di-TMS	N/A	143,73,121	464	2442	2439	trace	5.75	10.35	0.93
Eicosanoic acid, TMS	55530-70-6	73,117,132	384	2449	2447	trace	-	-	-
Diterpenoid C23H40O3Si	-	73,121,109	392	2449	-		4.08	-	-
Diterpenoid, TMS	-	73,143,156	462	2457	-	-	-	0.33	-
Diterpenoid, TMS	-	73,372,357	462	2462	-	-	-	0.34	-
Diterpenoid, TMS	-	73,341,356	462	2471	-	-	-	0.18	-
9-Pentacosene	N/A	97,83,57	350	2475	2470	0.19	-	-	-
7-Pentacosene	N/A	55,97,83	350	2482	2482	0.08	-	-	-
NN	-	285,73,286	-	2483	-	-	-	0.62	-
3-Pentadecenyl phenol, TMS	N/A	180,165,73	374	2483	-	-	-	-	trace
Hydroginkgol (hydrocardanol), TMS	N/A	180,179,165	376	2493	2492	0.07	-	-	0.08
Diterpenoid, TMS?	-	73,81,107	458?	2496	-	-	-	4.81	-
NN	-	73,81,75	-	2497	-	-	0.65	-	-
n-Pentacosane	629-99-2	57,71,43	352	2500	2500	0.04	-	-	0.09
Neoabietic acid, mono-TMS	N/A	135,73,121	374	2513	2508	-	7.03	1.39	0.07
NN (diterpenoid,TMS?)	-	143,73,81	302	2513	-	-	-	-	0.20
NN	-	191,73,234	460	2516	-	-	-	0.49	-
Diterpenoid, TMS	-	255,73,229	388	2520	-	-	-	0.33	-
Docosenol isomer 1, TMS?	-	75,73,96	396	2526	-	-	-	-	0.08
Docosenol isomer 2, TMS?	-	75,73,96	396	2531	-	-	-	-	0.06
NN	-	254,73,239	-	2534	-	-	0.83	0.92	-
Abieta-8,11,13-trien-19-oic acid, 7-hydroxy-,di-TMS	N/A	73,237,191	460	2543	-	-	0.31	1.11	-
Diterpenoid, TMS	-	73,121,120	466	2549	-	-	3.71	-	-
Imbricataloic acid, TMS	N/A	73,121,257	466	2550	2548	-	-	6.48	-

1-Docosanol, TMS	42449-18-3	383,73,43	-	2558	2556	-		-	0.02
NN	-	73,327,460	460?	2566	-	-	0.41	0.71	-
NN	-	73,327,175	-	2574	-	-	0.10	-	-
Diterpene acid, di-TMS (C26H46O3Si2?)	-	73,81,121	462	2588	-	-	0.15	1.01	-
Isocupressic acid, di-TMS	N/A	73,121,256	464	2602	2598	-	5.67	15.20	-
Diterpenoid, TMS	-	73,131,121	462	2617	-	-	0.46	-	-
NN	-	73,215,332	-	2621	-	-	0.54	-	-
Pinifolic acid, di-TMS	N/A	73,121,362	480	2641	2644	-	1.24	0.63	-
Diterpenoid, TMS	-	73,121,81	-	2646	-	-	0.66	1.89	-
NN (diteroenoid, TMS)	-	73,447,448	462	2650	-	-	0.86	1.04	-
3-[(9Z,12Z)-Heptadeca-9,12-dienyl]phenol	N/A	180,170,67	400	2665	2669	0.30	-	-	0.55
3-Heptadecenylphenol, TMS, isomer 1	-	180,402,55	402	2683	2685	0.05	-	-	0.55
3-Heptadecenylphenol, TMS, isomer 2	-	180,402,55	402	2689	-	0.15	-	-	0.10
3-Heptadecylphenol, TMS	N/A	180,404,179	404	2697	2693	-	-	-	0.14
n-Heptacosane	593-49-7	57,71,85	380	2700	2700	-	0.17	-	0.66
5-Pentadecenylresorcinol, di-TMS, isomer 1	-	268,73,269	462	2704	-	0.23	-	-	-
5-Pentadecenylresorcinol, di-TMS, isomer 2	-	268,73,269	462	2713	2717	0.61	-	-	0.23
5-Pentadecylresorcinol, di-TMS	N/A	268,464,73	464	2718	2717	-	-	-	1,00
NN	-	73,121,244	-	2719	-	-	0.20	-	-
NN	-	73,244,82	-	2720	-	-	-	0.62	-
NN	-	216,184,73	400?	2732	-	-	0.10	0.69	-
NN	-	73,121,81	-	2736	-		0.12	-	-
Dehydroabietic acid, 7α,15-hydroxy-, di-TMS	N/A	73,533,534	548	2750	2746	-	-	0.30	-
1-Tetracosanol, TMS	N/A	75,411,57	-	2754	2754	-	-	-	0.06
NN	-	131,73,75	-	2755	-	-	0.07	0.20	-
Dehydroabietic acid, 15-hydroxy-7-oxo-, TMS	N/A	73,459,460	-	2793	2790	-	-	0.23	-
NN	-	216,184,73	-	2805	-	-	0.06	0.39	-
(*Z*)-15-Tetracosenoic acid, TMS	N/A	73,75,117	438	2811	2809	-	-	-	0.09
5-Heptadecylresorcinol, di-TMS		268,73,75	478	2815	2812	-	-	-	trace
Squalene	111-02-4	69,81,41	410	2826	2828	0.10	-	-	trace
Ginkgolic acid, di-TMS	N/A	73,475,219	490	2843	2844	trace	-	-	0.06

Tetracosanoic acid, TMS	74367-37-6	117,73,75	440	2845	2845	trace	-	-	0.08
NN	-	229,73,347	-	2851	-	-	-	0.57	-
Hydroginkgolic acid, di-TMS	N/A	477,73,219	-	2859	-	0.22	-	-	0.30
Resorcinol, 5-(8,11)-heptadecadienyl-, di-TMS	N/A	268,73,269	488	2881	2881	3.04	-	-	4.29
NN	-	57,73,255	-	2891	-	-	0.05	-	-
Resorcinol, 5-heptadecatrienyl, di-TMS	N/A	268,73,180	486	2892	-	-	-	-	0.25
Resorcinol, 5-heptadecenyl, di-TMS, isomer 2	N/A	268,73,180	490	2893	2893	0.51	-	-	0.25
NN	-	274,73,275	-	2897	-	-	-	0.10	-
Resorcinol, 5-heptadecenyl, di-TMS, isomer 3	-	268,73,269	490	2898	2901	0.60	-	-	0.81
n-Nonacosane	630-03-5	57,71,85	408	2900	2900	-	1.61	-	0.81
Resorcinol, 5-heptadecenyl, di-TMS, isomer 4	-	268,73,180	490	2905	2906	2.32	0.04	-	1.40
Resorcinol, 5-heptadecyl, di-TMS	N/A	268,73,492	492	2913	2912	0.84	-	-	1.14
NN	-	83,73,55	-	2918	-	-	0.52	-	-
Squalene-like polyene	-	69,81,95	-	2929	-	0.04	-	-	-
Unidentified heptadecadienylresorcinol, TMS	-	268,73,269	488	2936	-	0.04	-	-	-
NN	-	148,73,57	-	2981	-	-	0.16	-	-
n-Triacontane	638-68-6	57,71,43	422	3000	3000	-	-	-	0.15
Salicylic acid, 6-heptadecadienyl, di-TMS	N/A	73,219,147	516	3031	3026	0.82	-	-	1.63
Salicylic acid, 6-heptadecenyl, isomer 1, di-TMS,	N/A	503,73,219	518	3043	3048	0.25	-	-	0.09
Hexacosanoic acid, TMS	N/A	117,73,453	468	3043	3043	-	-	-	0.18
Salicylic acid, 6-heptadecenyl, isomer 2, di-TMS,	N/A	503,73,219	518	3051	3055	0.21	-	-	0.39
Salicylic acid, 6-heptadecenyl, isomer 3, di-TMS,	N/A	503,73,219	518	3057	3059	0.97	-	-	0.61
n-Hentriacontane	630-04-6	57,71,85	436	3100	3100	-	0.66	-	8.48
Resorcynol, 5-nonadecenyl, di-TMS	N/A	268,73,269	518	3102	3100	0.59	-	-	-
Triterpenoid, TMS (lanostrerol-like)	-	69,393,189	498	3129	-	-	0.15	-	-
NN	-	143,73,173	-	3143	-	-	-	-	0.30
1-Octacosanol, TMS	959269-04-6	467,75,57	482	3148	3148	-	0.22		-
Triterpenoid, TMS (lanostrerol-like)	-	69,393,189	498	3190	-	-	0.35	-	-
Triterpenoid (β-amyrin-like), TMS	-	218,190,203	498	3204	-	0.26	1.01	-	-
Triterpenoid	-	69,363,73	398	3216	-	-	0.32	-	-
Triterpenoid, TMS (α-amyrin-like)	-	218,190,203	498	3230	-	0.18	-	-	-

Triterpenoid, TMS	-	218,190,203	398	3232	-	0.19	2.30	-	-
Triterpenoid, TMS	-	189,73	398	3240	-	-	0.33	-	-
Octacosanoic acid, TMS	1206693-36-8	73,117,75	496	3241	3241	-	-	-	0.18
Triterpenoid, C33H58OSI?	-	73,95,189	498	3241	-	0.19	-	-	-
3-Hydroxyergosta-7,22-diene, TMS	N/A	129,73,55	470	3251	3251	0.56	-	-	-
Eudesmin (lignan)	29106-36-3	165,177,151	386	3254	-	-	0.08	-	-
Salicylic acid, 6-(12-hydro,xyheptadecyl-), tri-TMS	N/A	73,219,531	546	3266	3261	0.45	-	-	0.38
Triterpenoid, TMS (lanosterol-like)	-	69,393,109	498	3266	-	-	0.11	-	-
Triterpenoid, TMS	-	69,81,95	498	3288	-	0.21	-	-	-
Triterpenoid	-	69,409,109	424	3283	-	-	2.08	-	-
n-Tritriacontane	630-05-7	57,71,85	464	3300	3300	-	0.07	190-	1.21
Dammaradien-3-one	N/A	109,205,69	424	3302	3305	0.40	-	-	-
Olean-12-en-3-one (β-amyrone)?	N/A	218,203,69	424	3310	-	-	0.64	-	0.76
Triterpenoid	-	218,203,95	424	3313	-	3.61	-	-	-
Triterpenoid, TMS	-	69,393,73	498	3315	-	-	0.66	-	-
Triterpenoid, TMS	-	229,95,73	-	3332	-	-	0.08	-	-
Triterpenol, TMS	-	109,69,95	498	3342	-	1.29	-	-	1.16
β-Amyrin, TMS	1721-67-1	218,203,73	498	3345	3345	9.01	0.69	0.11	5.08
1-Triacontanol, TMS	N/A	495,75,57	510	3347	3345	-	1.69	-	-
Triterpenoid C30H48O?	-	218,203,55	424	3356	-	-	1.02	-	-
Triterpenoid C30H48O?	-	109,95,205	424	3359	-	-	-	-	2.31
Triterpenoid C30H48O?	-	69,95,55	424	3364	-	-	-	-	1.37
Triterpenoid (isofucosterol?), TMS	-	129,55,69	484	3368	3363	10.25	-	-	-
α-Amyrin, TMS	N/A	218,189,73	498	3385	3378	8.73	0.40	0.07	3.96
Triterpenoid $C_{33}H_{56}OSi$		73,203,216	496	3395	-	-	0.25	-	-
Triterpenoid, TMS	-	189,109,73	498	3396	-	-	-	-	3.44
Cycloartenol, TMS	17608-55-8	69,73,95	498	3405	3406	-	-	0.21	11.54
Triterpenoid, TMS	-	69,199,73	-	3406	-	-	0.84	-	-
Lupeol, TMS	55804-02-9	73,109,189	496	3406	3407	9.87	-	0.07	-
Triterpenoid, TMS	-	69,95,73	-	3417	-	7.50	-	-	-
Triacontyl acetate	N/A	57,97,43	-	3418	-	-	1.44	-	-

Triterpenoid C31H50O, TMS?	-	95,55,69	438	3429	-	-	-	-	0.22
Triterpenoid TMS	-	203,73,190	-	3432		-	0.12	-	-
Bauer-7-en-3-one?	6895-55-2	245,95,55	424	3433	-	1.21	-	-	-
β-Amyrin acetate?	1616-93-9	218,203,69	468	3447	-	0.89	-	-	-
Triterpenoid, TMS	-	95,73,109	498	3447	-	-	-	-	0.53
Triacontanoic acid, TMS	1206693-37-1	495,75,496	510	3439	3441		0.09	-	-
9,19-Cyclolanostan-3-ol, 24-methylene-, TMS	N/A	73,95,69	512	3465	3968	-	0.05	-	1.22
Triterpenoid, TMS	-	73,95,69	512	3470	-	1.32	-	-	-
Triterpenoid, $C_{33}H_{58}OSi$	-	73,95,69	498	3478	-	-	0.06	-	-
Triterpenoid, $C_{33}H_{58}OSi$	-	189,109,73	498	3479	-	-	-	-	0.61
Triterpenoid, TMS		229,95,73	498	3481	-	1.53	-	-	-
Triterpenoid acetate?	-	69,95,109	468	3489	-	-	-	-	0.36
Triterpenol acetate?	-	218,189,95	468	3491	-	2.21	-	-	-
Dipterocarpol, TMS	N/A	199,69,73	514	3508	3504	0.60	-	-	1.87
Triterpenoid, TMS	-	73,422,203	-	3522	-	-	0.05	-	-
Triterpenoid, TMS	-	199,69,73	-	3539	-	1.39	-	-	0.96
Dotriacontanol, TMS	N/A	523,75,57	538	3545	3544		1.22	-	2.68
Triterpenoid, TMS	-	218,203,69	-	3561	-	0.54	-	-	-
Betulinol, di-TMS	N/A	73,189,203	-	3563	3564	-	-	-	0.19
Triterpenoid, TMS	-	203,190,73	-	3576	-	0.20	-	-	0.20
Lup-20(29)-en-28-al, 3-hydroxy, TMS?	N/A	73,189,95	512	3595	3610	-	-	-	0.20
Triacontyl butyrate?	N/A	89,71,57	508	3598	-	-	0.50	-	-
Triterpenoid, TMS	-	95,69,218	-	3607	-	1.96	-	-	-
Triterpenoid, TMS	-	131,215,73	-	3607	-	-	-	-	2.50
Triterpenoid, TMS	-	73,109,407	512	3607	-	-	0.20	-	-
Dotriacontyl acetate	23811-94-1	57,43,97	508	3619	3615	-	1.38	-	0.90
Triterpenoid, TMS	-	131,215,73	-	3635	-	1.18	-	-	0.44
Triterpenoid, TMS	-	131,73,75	-	3645	-	2.53	-	-	0.71
Triacontanol metacrylate?	N/A	87,86,69	506-	3660	-	-	0.25	-	-
Triterpenoid, TMS	-	69,73,147	-	3662	-	0.36	-	-	0.28
NN	-	357,73,358	-	3681	-	-	0.27	-	-

NN	-	73,95,121	-	3695	-	-	0.53	-	-
Tetratriacontanol, TMS	N/A	551,75,57	566	3741	3742	-	0.19	-	-
NN	-	73,75,552	-	3743	-	1.07	-	-	-
Tetratriacontanol, TMS	N/A	551,75,73	566	3746	3743	-	-	-	3.72
Triterpenoid, TMS	-	95,73,55	526	3753		-	0.36	-	-
Triterpenoid, TMS	-	73,95,107	-	3761	-	0.62	-	-	0.70
NN	-	73,95,107	-	3770	-	0.83	-	-	1.26
NN	-	73,197,107	-	3785	-	-	-	-	0.92
iso-Mangiferolic acid, di-TMS	-	73,95,175	600	3793	3793	2.41	-	-	2.06
Dotriacontanol butyrate?	-	89,71,57	-	3799	-	-	0.47	-	-
NN	-	73,143,197		3800	-	-	-	-	0.23
NN	-	73,219,95	-	3804	-	-	-	-	0.28
Tetratriacontanol acetate?	-	57,97,43	-	3819	-	-	0.30	-	3.15
NN	-	73,57,97	540	3822	-	2.25	-	-	-
NN	-	161,117,57	596?	3832	-	-	0.64	-	-
NN	-	73,95,107	-	3850	-	1.06	-	-	1.24
Dotriacontanol metacrylate?	N/A	87,86,69	534	3862	-	-	0.23	-	-
NN	-	95,73,107	526	3874	-	-	trace	-	-
Mangiferonic acid, TMS	-	73,95,121	526	3878	3877	2.46	-	-	1.94
NN	-	73,95,107	-	3897		-	0.13	-	-
Mangiferolic, TMS	-	73,95,125	600	3902	3902	3.81	-	-	5.01
1-Hexatriacontanol acetate?	N/A	73,579,57	594	3940	-	0.76	-	-	1.13
NN	-	73,147,95	-	3968	-	0.54	-	-	0.65
Aliphatic acid acetate	-	57,43,97	-	4025	-	1.87	-	-	0.35
NN	-	161,117,57	-	4035	-	-	0.50	-	0.55
NN		73,147,95	-	4067	-	0.57	-	-	0.81
Tetracosyl hexadecanoate	N/A	257,57,43	592	4294	-	0.27	-	-	0.16

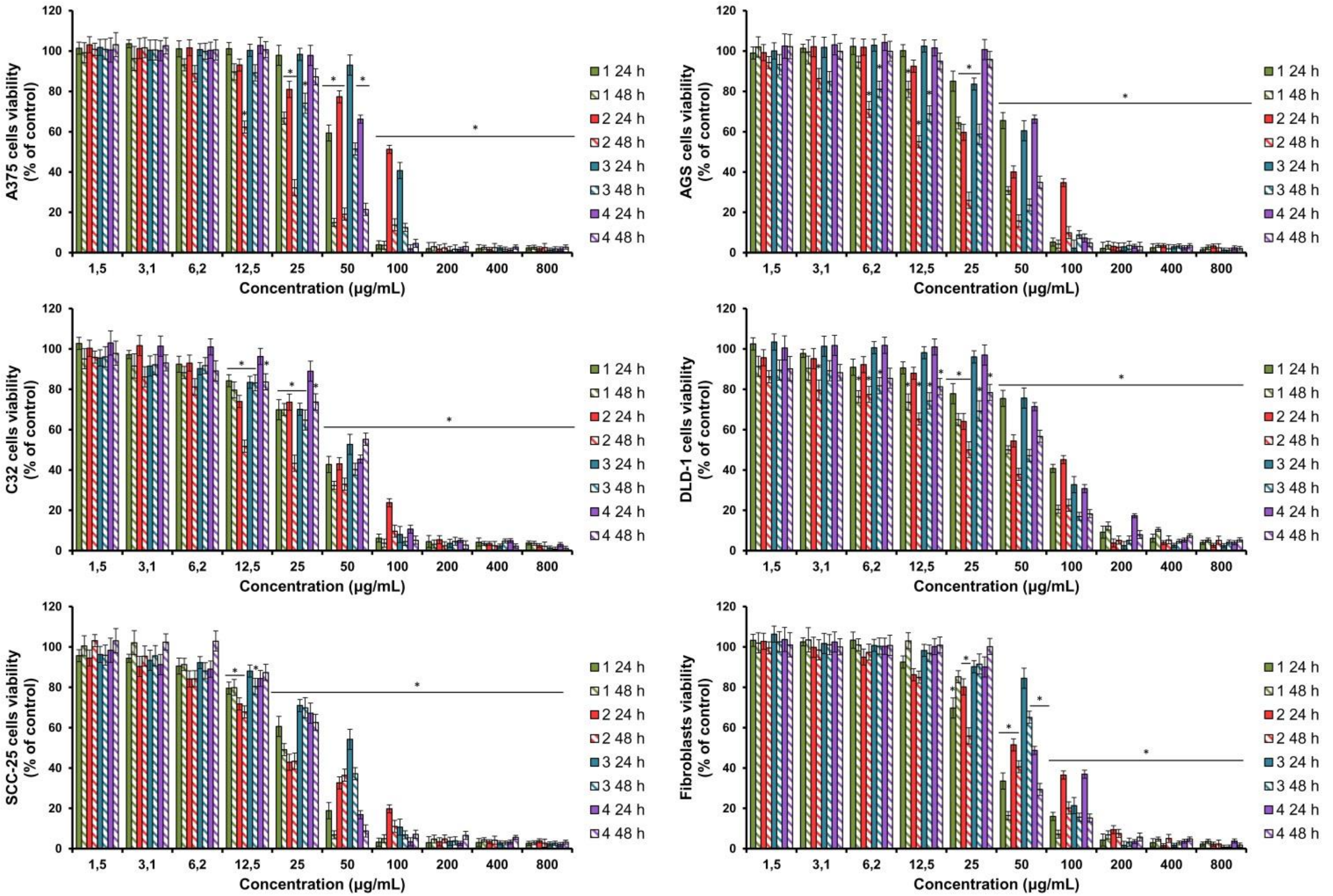

Figure S1. The viability of A375, C32, SCC-25, AGS, DLD-1 cells and fibroblasts treated with four propolis extracts for 24 h and 48 h. Data are presented as mean ± standard error of the mean (SEM) from three independent experiments.

* $p < 0.05$ compared to control group.

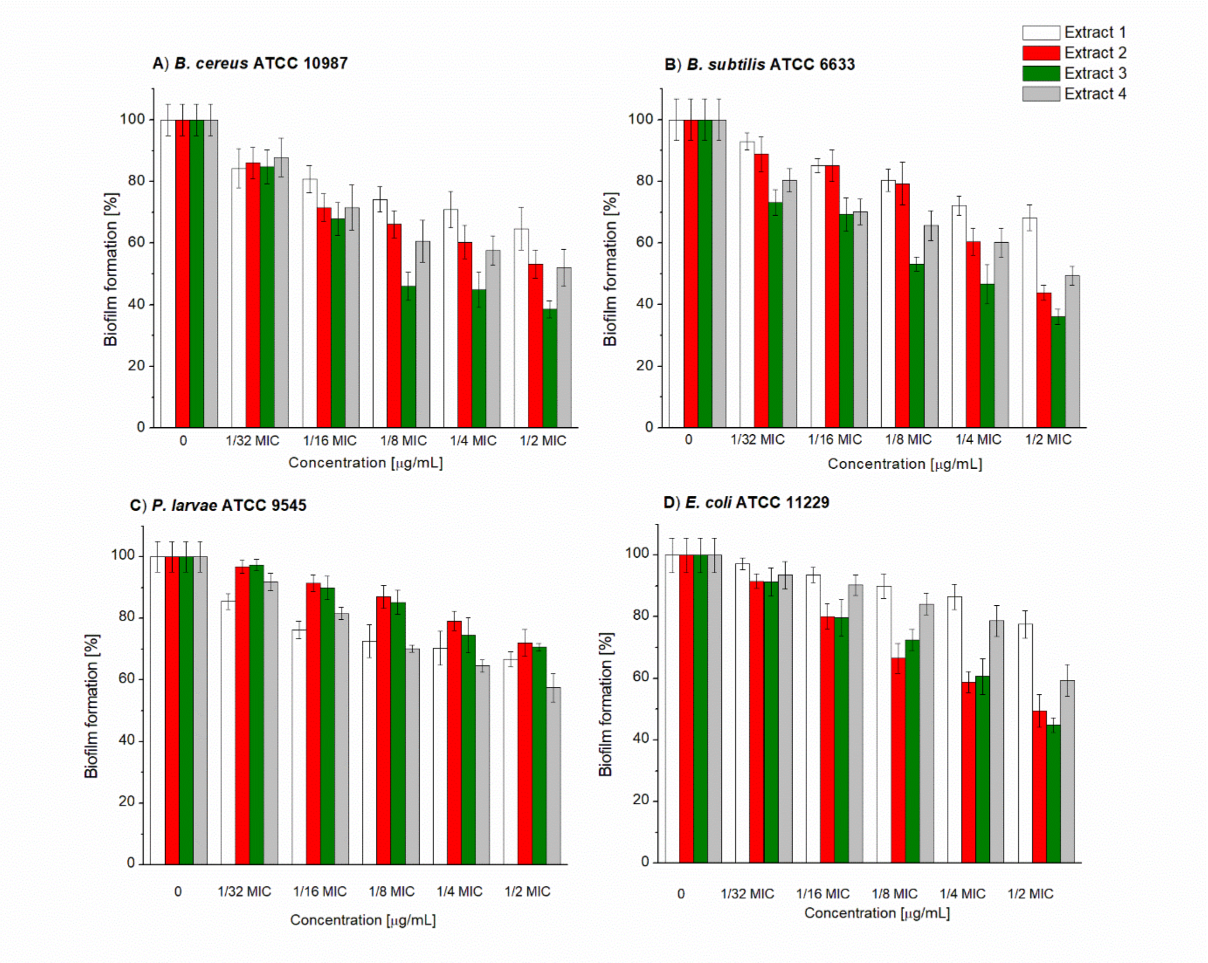

Figure S2. The effect of propolis extracts on biofilm formation
